# Expression of CD133 in the cytoplasm is associated with cancer progression and poor prognosis in gastric cancer

**DOI:** 10.1007/s10120-013-0255-9

**Published:** 2013-04-05

**Authors:** Kousuke Hashimoto, Keishiro Aoyagi, Taro Isobe, Kikuo Kouhuji, Kazuo Shirouzu

**Affiliations:** Department of Surgery, Kurume University School of Medicine, 67 Asahi-machi, Kurume, Fukuoka 830-0011 Japan

**Keywords:** Gastric cancer, CD133, Prognosis, HIF-1α

## Abstract

**Background:**

CD133 is one of the most important stem cell markers in solid cancers. Some recent reports have described a possible relationship between CD133 and hypoxia-inducing factor-1-alpha (HIF-1α). The aim of this study was to clarify the clinical role of CD133 expression in gastric cancer and to investigate the correlation between CD133 expression and HIF-1α expression.

**Methods:**

We studied 189 gastric cancer patients who underwent gastrectomy at Kurume University Hospital. CD133 and HIF-1α expression was examined using immunohistochemical staining. Fifty-six cases were CD133 positive, and they were divided into two expression types: luminal expression of the gland and cytoplasmic expression. We investigated the relationship among CD133 expression types, clinicopathological variables, prognosis, and HIF-1α expression.

**Results:**

When comparing clinicopathological variables, expression of CD133 in the cytoplasm was related to metastasis and tumor progression. However, this relationship was not observed with luminal expression of the gland type. The survival rate in patients with cytoplasmic CD133 expression was significantly worse than that in the CD133-negative group. This relationship was observed in the survival rate of the adjuvant chemotherapy group and the curative resection group. Multivariate analysis revealed that the expression of CD133 in the cytoplasm was an independent prognostic factor in gastric cancer. Regarding the correlation between CD133 expression and HIF-1α expression, the HIF-1α positive rate was lower in patients with CD133 luminal expression of the gland type and higher in patients with cytoplasmic expression of CD133.

**Conclusion:**

Gastric cancer cells with CD133 expression in the cytoplasm were cells with high potential for malignancy, and this phenotype was associated with cancer progression, chemotherapy resistance, recurrence, and poor prognosis. Cytoplasmic expression of CD133 may be a useful prognostic marker in gastric cancer. Significant correlation was observed between HIF-1α expression and the immunohistochemical staining pattern of CD133.

## Introduction

Since cancer stem cells (CSCs) in solid cancers [1, 2] were first reported in the early half of the 2000s, the establishment of a treatment targeting CSCs for radical cure of cancer has become an important goal. Therefore, the search for markers to isolate CSCs and characterize cells isolated with these markers has been active throughout the world.

CD133 is a 120-kDa glycoprotein with five transmembrane domains and is a CSC marker. Despite various theories, the biological function of CD133 is still not well understood. Originally, CD133 was known as a surface marker of hematopoietic stem cells and progenitor cells, but CD133 has also recently been reported as a marker of CSCs in solid cancers such as brain tumors [2], lung cancer [3], liver cancer [4], colon cancer [5, 6], pancreatic cancer [7], and prostate cancer [8]. In addition, in lung cancer [9], breast cancer [10], hepatocellular carcinoma [11], colon cancer [12], and pancreatic cancer [13], CD133 expression has been reported to be strongly related not only to tumor progression, but also to treatment resistance.

However, despite the large number of patients with gastric cancer in Japan, CSCs in gastric cancer have not been definitively reported, and few studies evaluating CD133 expression have been reported. In highly advanced gastric cancer and recurrent gastric cancer, compared to colon cancer, there is still no effective treatment, and survival rates remain low. Therefore, identification of gastric CSCs and establishment of treatment will be highly important in future gastric cancer therapy.

Regarding CSCs, a hypoxic environment has recently been shown to be necessary to maintain CSCs [14]. Hypoxia-inducing factor-1alpha (HIF-1α) is a downstream molecule in the mammalian target of rapamycin signaling pathway, is induced by hypoxemia, and acts as a transcription factor. HIF-1α has attracted attention as a factor that regulates CD133 expression, and the relationship between CD133 expression and HIF-1α expression has been investigated in various solid cancers. Most studies have shown a correlation between CD133 expression and HIF-1α expression [15, 16], but interestingly, downregulation of CD133 expression by HIF-1α expression in a gastric cancer cell line has also been reported [17].

In this study, we investigated the clinicopathological role of CD133 expression in gastric cancer by immunostaining clinical specimens from gastric cancer patients. We also evaluated the relationship between CD133 expression and prognosis of gastric cancer. In addition, we examined the relationship between CD133 expression and HIF-1α expression using immunohistological staining of gastric cancer tissue specimens.

## Materials and methods

### Patients

Paraffin specimens from 189 gastric cancer patients who underwent gastrectomy between January 2004 and August 2006 at Kurume University Hospital were selected. Double cancer, multiple cancer, mucosal cancer, postendoscopic mucosal resection, and endoscopic submucosal dissection cases were excluded from this study. Histopathological characteristics and each classification are defined in the Japan Classification of Gastric Carcinoma (14th edition) [18]. The patient characteristics are shown in Table 1. Mean age was 66 years, and there were more men than women. Regarding histological type, we divided the patients into a differentiated group (tub1, tub2, pap), and an undifferentiated group (por1, por2, sig, muc). The residual tumor (R) was defined as R0, no residual tumor; R1, microscopic residual tumor (positive resection margin or CY1); or R2, macroscopic residual tumor. None of the patients had received preoperative adjuvant therapy, but 82 patients had received postoperative adjuvant therapy.

**Table 1:** Patient information

Characteristic	Number of patients (*n* = 189)
Age (mean ± SD), years	66 ± 11
Gender (male/female)	133/56
Tumor size (mean ± SD), mm	66 ± 38
Histological type^a^ (differentiated/undifferentiated)	81/108
Stage (I/II/III/IV)	62/41/52/36
Surgery	
Total gastrectomy (include remnant gastrectomy)	72
Distal gastrectomy	99
Proximal gastrectomy	11
Segmental gastrectomy	7
R (residual tumor)^b^, 0/1/2	144/11/34
Adjuvant chemotherapy (−/+)	107/82
Regimen	
TS-1^c^	37
Oral anticancer drug except TS-1	25
TS-1 + continuous infusion anticancer drug	15
Details unknown	5

*SD* standard deviation

^a^Differentiated (tub1, tub2, pap); undifferentiated (por1, por2, sig, muc)

^b^R0, nonresidual tumor; R1, microscopic residual tumor (positive resection margin or CY1); R2, macroscopic residual tumor

^c^TS-1 is an oral anticancer drug containing a 5-fluorouracil derivative (tegafur)

This study was authorized in advance by the Ethics Committee of Kurume University (study number 11040).

### CD133 staining

CD133 expression and HIF-1α expression were examined with immunohistochemical staining. Surgical specimens were fixed in 10 % formaldehyde, embedded in paraffin, and cut into 4-μm-thick sections. We chose the most invasive section from the gastric cancer tumor. Sections were deparaffinized in xylene and rehydrated in a graded series of ethanol. Slides were heated at 120 °C in an autoclave in 10 mM sodium citrate (pH 6.0) for 10 min and then cooled to room temperature. After blocking with 10 % horse serum, the sections were incubated overnight at 4 °C with mouse monoclonal anti-CD133 antibody [Milteny Biotec, Auburn, CA, USA; diluted 1:100 in phosphate-buffered saline (PBS)]. After washing, sections were overlaid with secondary antibody (VECTASTAIN elite ABC kit Universal; Vector Laboratories, Burlingame, CA, USA) for 30 min at room temperature. Sections were incubated in 3.0 % hydrogen peroxide in PBS for 30 min to block endogenous peroxidase activity. The reaction was developed using avidin–biotin–peroxidase complex. The peroxidase reaction was developed with 3-amino-9-ethylcarbazole, and sections were counterstained with hematoxylin. Colon cancer sections were used as a positive control. Negative control sections (isotype control) were incubated with normal mouse serum instead of the primary antibody.

### HIF-1α staining

Isobe et al. [19] have reported detailed methods for immunohistochemical staining of HIF-1α. Briefly, 4-μm-thick sections were cut from archival formalin-fixed paraffin-embedded tissue blocks. Slides were irradiated at 99 °C in a microwave oven for 30 min in 10 mM citrate buffer (pH 9.0) and cooled to room temperature. The sections were incubated overnight at 4 °C with rabbit polyclonal anti-HIF-1α antibody H206 (Santa Cruz Biotechnology, Santa Cruz, CA, USA; diluted 1:100 in PBS), and reactions were developed using the same method as CD133 staining.

### Double immunohistochemical staining

To clarify the localization of CD133 in gastric cancer cells, double immunohistochemical staining with anti-CD133 antibody and anti-cytokeratin 8 antibody was performed in some cases. The reaction was developed using anti-mouse poly-alkaline phosphatase (AP) and anti-rabbit poly-horseradish peroxidase (HRP) polymerization technology. We used a cocktail of primary antibodies containing the same anti-CD133 antibody (diluted 1:100 in PBS) and rabbit monoclonal anti-cytokeratin 8 antibody (Abcam, Cambridge, UK; diluted 1:50 in PBS). After incubating overnight at 4 °C, sections were overlaid with secondary antibody (MACH2 Double Stain 1 Mouse-AP ± Rabbit-HRP; Biocare Medical, Concord, CA, USA). The alkaline phosphatase reaction for the anti-CD133 antibody was developed with Vulcan fast red chromogen (Biocare Medical; Vulcan Fast Red Chromogen Kit2), and the peroxidase reaction for the cytokeratin 8 antibody was developed with 3,3′-diaminobenzidine (DAB).

In addition, to confirm both CD133 and HIF-1α expression in gastric cancer, double immunohistochemical staining was performed in some cases. The reaction was developed using AP and HRP polymerization technology as described above. We used a cocktail of primary antibodies containing the same anti-CD133 antibody (diluted 1:100 in PBS) and the same anti-HIF-1α antibody (diluted 1:100 in PBS). The secondary antibody and the alkaline phosphatase reaction were developed using the same methods as described for the CD133 + cytokeratin 8 staining. The peroxidase reaction for the anti-HIF-1α antibody was developed with DAB.

### Statistical analysis

For comparison of categorical data, the chi-square test and Fisher’s exact test were used. The overall survival rate was calculated using Kaplan–Meier analysis, and differences between the groups were compared using the log-rank test. For multivariate analysis, prognostic factors were analyzed using Cox’s proportional hazard model. All statistical analysis was performed using statistical software (JMP 9.0; SAS, Cary, NC, USA). *P* < 0.05 was considered statistically significant.

## Results

### Immunohistochemical findings

**Fig. 1:**
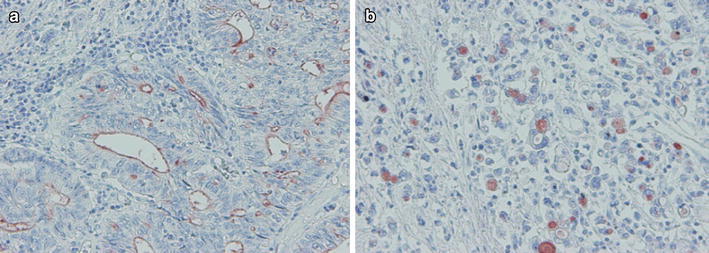
Two general CD133 expression types were observed in gastric cancer. **a** Luminal expression in the gland (L-type). **b** Expression in the cytoplasm (C-type). **a**, **b** ×200

Positive expression for CD133 was observed only in cancer cells. Two general expression types were observed in gastric cancer (Fig. 1a, b). In differentiated gastric cancer in particular, we observed luminal expression of the gland (defined as L-type) as in colon cancer [20], and in undifferentiated gastric cancer in particular, we saw expression in the cytoplasm (defined as C-type) as in pancreatic cancer [21]. In some cases, we saw both L-type and C-type on the same section. We defined the more dominant expression type as the main type in the specimens. CD133 expression was evaluated in 1,000 cancer cells in high-power fields. The frequency of CD133 expression cells in 1,000 cancer cells was 0–18.3 %. In 6 cases, we stained some sections from the gastric cancer tumor, but there were no differences in the CD133 expression frequency and expression type (data not shown). CD133 expression was observed in 128 of 189 cases (67.7 %). We defined CD133 positive as more than 5 % positively stained cancer cells. Of the 189 total cases, we observed 56 CD133-positive cases (29.6 %), 33 L-type cases (17.4 %), and 23 C-type cases (12.1 %).

**Fig. 2:**
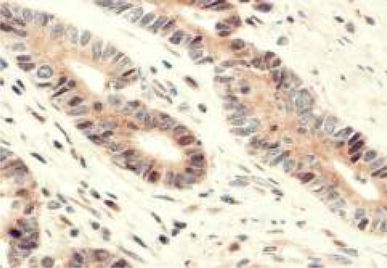
Expression of hypoxia-inducible factor (HIF)-1α was seen in the nucleus of cancer cells. ×400

HIF-1α expression was observed in the nucleus of cancer cells (Fig. 2). Concomitant cytoplasmic staining was ignored because HIF-1α protein is functionally active in the nucleus. We defined HIF-1α as positive when more than 5 % of cancer cells showed positive nuclear expression. Of the 189 total cases, 107 (56.6 %) were HIF-1α positive.

**Fig. 3:**
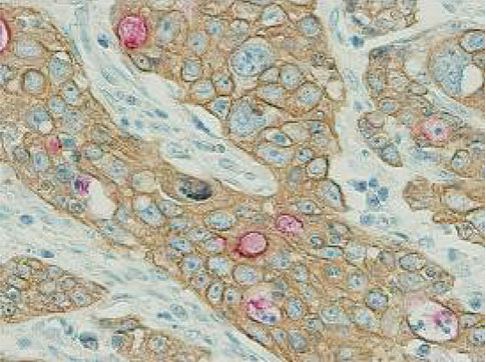
Double immunohistochemical staining with anti-CD133 antibody (*red*) and anti-cytokeratin 8 antibody (*brown*). In the C-type, CD133 was mainly expressed in the intracytoplasmic lumen (ICL). ×400

Cytokeratin 8 is a cytoplasmic marker of adenocarcinoma including gastric cancer. Double immunohistochemical staining with anti-CD133 antibody and anti-cytokeratin 8 antibody revealed that cytoplasmic expression of CD133 was present mainly in the intracytoplasmic lumen (ICL) (Fig. 3). The occurrence of ICL, which is well known in breast cancer, has been reported in gastric cancer [22].

**Fig. 4:**
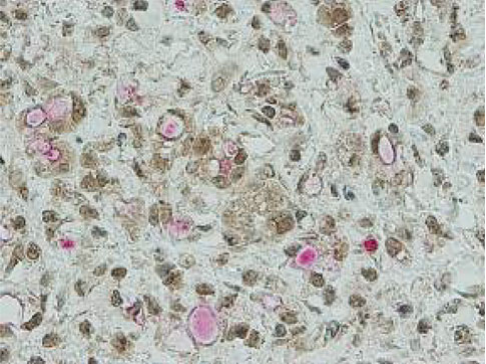
Gastric cancer was confirmed to express both CD133 (*red*) and HIF-1α (*brown*). We observed HIF-1α expression in the nucleus in the C-type. ×400

Gastric cancer specimens were confirmed to express both CD133 and HIF-1α. Moreover, HIF-1α expression in the nucleus tended to be present more often in C-type than L-type cases (Fig. 4).

### Clinicopathological significance of CD133 expression

First, we investigated the clinicopathological role of CD133 expression in gastric cancer. Table 2 shows the relationship between expression of CD133 and clinical variables. We divided our cases into three groups: CD133 negative, L-type positive, and C-type positive. The rates of lymph node metastasis, peritoneal dissemination, vascular invasion, and advanced stage tended to be higher in the C-type-positive group than in the other two groups, and significant differences among these three groups were observed. No correlation was observed in the L-type-positive group. Thus, we hypothesized that CD133 expression in the cytoplasm was related to cancer progression.

**Table 2:** Comparison of clinical variables between CD133-positive and CD133-negative cases

Variable	CD133 expression			
	Negative (*n* = 133)	Positive		*P* value
		L-type positive (*n* = 33)	C-type positive (*n* = 23)	
Age (years)				
<70	74 (56 %)	15 (45 %)	11 (48 %)	0.503
≥70	59 (44 %)	18 (54 %)	12 (52 %)	
Gender				
Male	88 (66 %)	31 (94 %)	14 (61 %)	0.004
Female	45 (33 %)	2 (6 %)	9 (39 %)	
Macroscopic type				
0	43 (32 %)	9 (27 %)	5 (21 %)	0.176
1, 2	32 (24 %)	13 (39 %)	4 (17 %)	
3, 4	58 (43 %)	11 (33 %)	14 (60 %)	
Diameter (mm)				
<70	73 (55 %)	22 (67 %)	10 (43 %)	0.219
≥70	60 (45 %)	11 (33 %)	13 (56 %)	
Region				
U	33 (25 %)	12 (36 %)	7 (30 %)	0.230
M	37 (27 %)	3 (9 %)	6 (26 %)	
L	63 (47 %)	18 (54 %)	10 (43 %)	
T classification				
1, 2	59 (44 %)	12 (36 %)	7 (30 %)	0.374
3, 4	74 (55 %)	21 (63 %)	16 (69 %)	
N classification				
0	71 (53 %)	16 (48 %)	4 (17 %)	0.006
1, 2, 3	62 (46 %)	17 (51 %)	19 (82 %)	
Lymph node metastasis number				
0	71 (53 %)	16 (48 %)	4 (17 %)	0.011
1–6	23 (17 %)	10 (30 %)	7 (30 %)	
≥7	39 (29 %)	7 (21 %)	12 (52 %)	
M classification				
0	114 (86 %)	27 (82 %)	13 (57 %)	0.003
1	19 (14 %)	6 (18 %)	10 (43 %)	
H classification				
0	132 (99 %)	29 (87 %)	20 (87 %)	0.001*
1	1 (1 %)	4 (12 %)	3 (13 %)	
P classification				
0	121 (91 %)	32 (97 %)	16 (70 %)	0.024*
1	12 (9 %)	1 (3 %)	7 (30 %)	
CY classification				
0	124 (93 %)	32 (97 %)	20 (87 %)	0.521*
1	9 (7 %)	1 (3 %)	3 (13 %)	
Stage				
I, II	80 (60 %)	17 (52 %)	6 (26 %)	0.009
III, IV	53 (39 %)	16 (48 %)	17 (73 %)	
Histological type^a^				
Differentiated	51 (38 %)	25 (75 %)	5 (22 %)	<0.001
Undifferentiated	82 (61 %)	8 (24 %)	18 (78 %)	
Stroma				
med, int	104 (78 %)	31 (94 %)	14 (61 %)	0.011
sci	29 (21 %)	2 (6 %)	9 (39 %)	
INF				
a, b	83 (62 %)	29 (87 %)	11 (48 %)	0.004
c	50 (37 %)	4 (12 %)	12 (52 %)	
ly				
0, 1	58 (44 %)	13 (39 %)	4 (17 %)	0.059
2, 3	75 (56 %)	20 (60 %)	19 (82 %)	
v				
0, 1	125 (94 %)	30 (91 %)	17 (74 %)	0.004*
2, 3	8 (6 %)	3 (9 %)	6 (26 %)	

Histopathological characteristics and each classification are defined according to the Japan Classification of Gastric Carcinoma (14th edition)

*L-type* luminal expression of the gland type, *C-type* expression in the cytoplasm type

^a^Differentiated (tub1, tub2, pap); Undifferentiated (por1, por2, sig, muc)

* Calculated with Fisher’s exact test

### Prognostic significance of CD133 expression

**Fig. 5:**
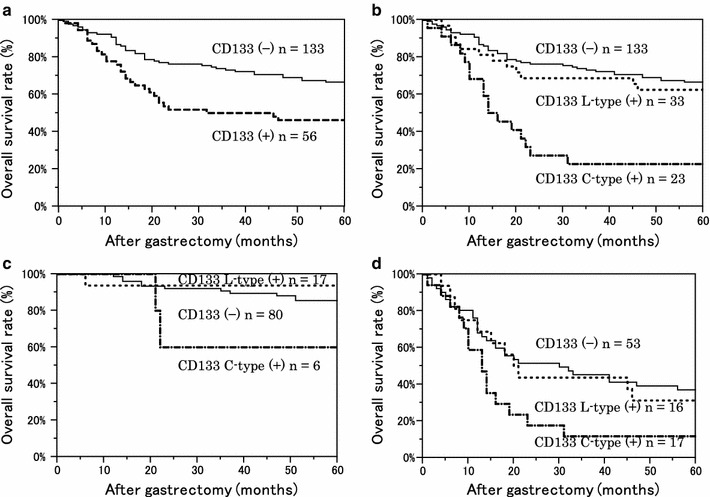
Kaplan–Meier survival curves of 189 patients with gastric cancer, stratified by CD133 expression. **a** The 5-year overall survival (OS) rate of the CD133-negative (−) group was 66.6 %; for the positive (+) group, OS was 46.3 % (*P* = 0.004 with the log-rank test). **b** Divided into two expression types, the 5-year OS rate of the L-type (+) was 62.5 %; for the C-type (+), the OS was 22.7 %. *P* = 0.603 between CD133 (−) and L-type (+), and *P* < 0.001 between CD133 (−) and C-type (+). **c** Survival curves of stage I/II. The 5-year OS rate of C-type (+) was 60.0 %. There was no significant difference in the survival curves between CD133 (−) and C-type (+) (*P* = 0.191). **d** In stage III/IV, the 5-year survival rate of C-type (+) was 11.7 %. There was a significant difference in the survival curves between CD133 (−) and C-type (+) (*P* = 0.017)

Figure 5 shows the relationship between prognosis and CD133 expression. The overall survival rate for the CD133-positive group was significantly worse than that in the CD133-negative group (Fig. 5a). When the CD133-positive cases were divided into two expression types, the C-type-positive group showed significantly worse survival (Fig. 5b). This tendency was present regardless of the stage (Fig. 5c, d). For prognosis with multivariate analysis, we controlled for T, N, and P factors, which were strong prognostic factors selected with backward stepwise regression, and for pathological type and HIF-1α expression, which may be confounding factors. Multivariate analysis revealed that CD133 C-type positive was an independent prognostic factor in gastric cancer (Table 3).

**Table 3:** Multivariate analysis of the relationship between CD133 expression type and overall survival

Analysis	CD133 expression		
	Negative	L-type positive	C-type positive
Univariate			
HR	1	1.18	3.62
95 % CI	–	0.59–2.18	1.99–6.29
*P* value	–	0.604	<0.001
Multivariate			
Model 1^a^			
HR	1	1.16	3.59
95 % CI	–	0.57–2.19	1.97–6.24
*P* value	–	0.654	<0.001
Model 2^b^			
HR	1	1.02	1.92
95 % CI	–	0.50–1.94	1.02–3.45
*P* value	–	0.945	0.041
Model 3^c^			
HR	1	1.06	1.87
95 % CI	–	0.52–2.05	1.00–3.39
*P* value	–	0.846	0.049

We confined the variables that were incorporated into the analysis to nine from the number of events

*HR* hazard ratio, *CI* confidence interval

^a^Model 1 was analyzed using the Cox proportional hazard model while controlling for age (<70 years, ≥70 years) and gender

^b^Model 2 includes model 1 variables plus T (1, 2/3, 4), N (0/1, 2, 3), and P (0/1) classifications

^c^Model 3 includes model 2 variables + HIF-1α expression and histological type (differentiated, undifferentiated)

### Relationship between CD133 expression and chemotherapy resistance and recurrence

**Fig. 6:**
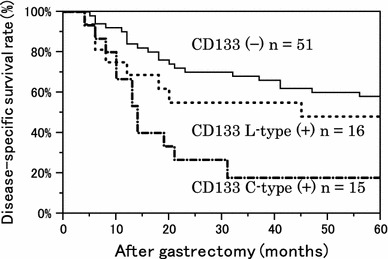
Kaplan–Meier survival curves of 82 patients who underwent adjuvant chemotherapy (adjuvant chemotherapy included all chemotherapeutic regimens and durations of administration). *P* = 0.341 between CD133 (−) and L-type (+); *P* < 0.001 between CD133 (−) and C-type (+)

**Fig. 7:**
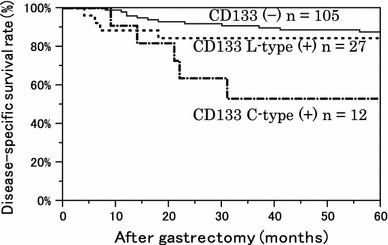
Kaplan–Meier survival curves of 144 patients with R0 status (R0 is a curative resection with negative resection margins). *P* = 0.563 between CD133 (−) and L-type (+), and *P* = 0.013 between CD133 (−) and C-type (+)

Furthermore, we investigated the relationship between CD133 expression and chemotherapy resistance and recurrence. We selected patients who underwent adjuvant chemotherapy and a curative resection, and disease-specific survival rate curves were compared among the CD133 expression types. In both the adjuvant chemotherapy group and the curative resection group, the survival rate curve of the C-type-positive group was significantly worse than that in the other groups (Figs. 6, 7).

### Correlation between CD133 expression and HIF-1α expression

**Fig. 8:**
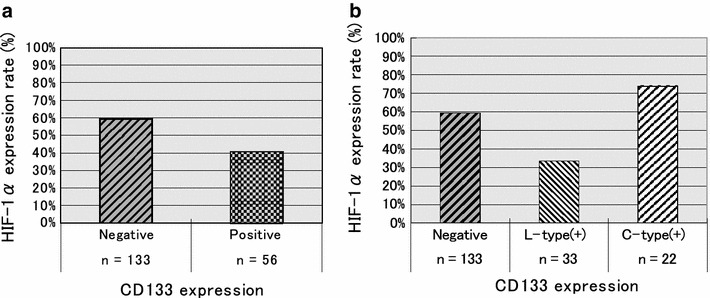
Correlation between CD133 expression and HIF-1α expression. **a** HIF-1α expression rate was 59.4 % in the CD133-negative group and 40.6 % in the CD133-positive group. There was no significant difference between the two groups (*P* = 0.234). **b** The cases were divided into two expression types. The HIF-1α expression rate was 33.3 % in the L-type-positive group and 73.9 % in the C-type-positive group. *P* = 0.041 among the three groups

Finally, we examined the correlation between CD133 expression and HIF-1α expression. There was no significant difference in HIF-1α expression between the CD133-positive and CD133-negative groups. However, when we analyzed the groups according to expression type, the HIF-1α-positive rate was lower in L-type and higher in C-type cases. We found a significant difference in the HIF-1α expression rate among these three groups (Fig. 8a, b).

**Fig. 9:**
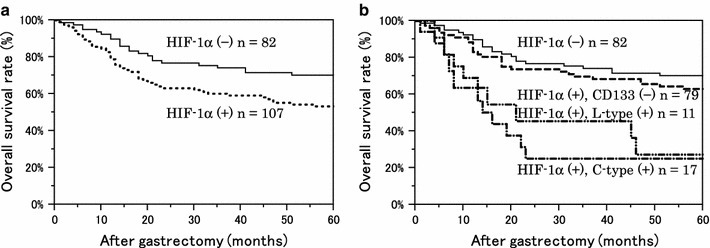
Kaplan–Meier survival curves of 189 patients with gastric cancer, stratified by HIF-1α expression and CD133 expression. **a** The 5-year OS rate was 70.2 % for the HIF-1α-negative (−) group and 53.2 % for the positive (+) group. *P* = 0.017 with the log-rank test. **b** Stratified by CD133 expression type, the 5-year OS rate of the HIF-1α (+)/CD133 (−) group was 63.0 %. For the HIF-1α (+)/L-type (+) group, OS was 27.2 %. For the HIF-1α (+)/C-type (+) group, OS was 25.1 %. *P* = 0.332 between the HIF-1α (−) and HIF-1α (+)/CD133 (−) groups, *P* = 0.001 between the HIF-1α (−) and HIF-1α (+)/L-type groups, and *P* < 0.001 between the HIF-1α (−) and HIF-1α (+)/C-type (+) groups

We have previously reported that HIF-1α expression is a poor prognostic factor in gastric cancer [19]. In the present study, the overall survival rate curve of the HIF-1α-positive group was significantly worse than that in the HIF-1α-negative group (Fig. 9a). However, if we divided the HIF-1α-positive group according to CD133 expression, both the CD133-positive and the HIF-1α-positive groups showed poor prognosis. Interestingly, a similar result was seen without regard to the CD133 expression type (Fig. 9b).

## Discussion

We found that CD133 protein expression in gastric cancer clinical specimens was the same as that in other solid cancers. CD133 expression was detected in about 30 % of cases. This expression could be broadly divided into two types: glandular-luminal cell membrane surface expression (luminal expression, L-type) and cytoplasmic expression (C-type). Luminal expression was more common in differentiated gastric cancer, and cytoplasmic expression was more common in undifferentiated gastric cancer, and both expression types were seen in some tissue sections. Ishigami et al. [23] and Zhao et al. [24] also evaluated CD133 expression in gastric cancer using clinical specimens, and both reported two types of staining results, similar to our findings. Originally, luminal expression of CD133 was reported in colorectal cancer, and cytoplasmic expression of CD133 was reported in pancreatic cancer [20, 21]. However, both expression types have recently been reported at the same time in colon cancer and in hepatocellular carcinoma [25, 26].

These reports also support the possibility of two types of CD133 expression in gastric cancer. A definitive difference was reported in another article about the expression of CD133 in gastric cancer [23]. Ishigami et al. [23] evaluated the overall CD133 expression in gastric cancer, without dividing the cases into expression types. On the other hand, in our study, we focused on these two expression types, with the analysis divided for each expression type. Ishigami et al. [23] reported that CD133 expression in gastric cancer is a risk factor for tumor progression, prognosis, depth of invasion, and lymph node metastases. In our study, the CD133 cytoplasmic expression was certainly related to tumor progression, primarily metastasis such as lymph node metastasis, peritoneal dissemination, and vascular invasion. With multivariate analysis, cytoplasmic CD133 expression was an independent prognostic factor. However, we did not find a definitive relationship between luminal CD133 expression and the degree of malignancy.

Sasaki et al. [25] reported that in hepatocellular carcinoma, cytoplasmic CD133 expression, rather than membranous expression, is related to the degree of malignancy and prognosis. In rectal cancer as well, cytoplasmic CD133 expression is related to local recurrence and prognosis in a group that underwent preoperative chemotherapy and radiotherapy [27]. These reports suggest that cytoplasmic CD133 expression alone may also be involved in the degree of malignancy of gastric cancer. It is widely known that some proteins gain biological function based on their site of expression. CD133 may be one of these types of proteins.

What is the significance of CD133 luminal expression? In an interesting report, Fukamachi et al. [28] used fluorescence-activated cell sorting (FACS) analysis of gastric cancer tissue and reported that loss of CD133 expression on the glandular luminal surface may be related to gastric tumor progression. However, our study results suggest that release of CD133 from the cytoplasm of undifferentiated gastric cancer cells into the glandular lumen may promote gland duct formation. In any case, CD133 expression likely plays some role in differentiation, as shown by Yang et al. [29].

Therefore, are CD133-expressing cancer cells actually CSCs? Characteristics of CSCs include tumorigenicity, treatment resistance, and tumor recurrence. Among these, tumorigenicity would be difficult to demonstrate in this study. Therefore, we investigated treatment resistance and tumor recurrence. The survival rate in the postoperative adjuvant chemotherapy group and the curative resection group was regarded as an indicator of treatment resistance and recurrence. Among these patients, the survival rate in the cytoplasmic CD133 expression group was significantly lower than in the other groups. This finding showed that the cytoplasmic CD133 expression group acquired treatment resistance and was more likely related to tumor recurrence. In summary, cancer cells with cytoplasmic CD133 expression were related to tumor progression (primarily metastases) and prognosis, and were associated with more undifferentiated tumors, treatment resistance, and more likely recurrence. These results suggested that these cells may have CSC-like characteristics.

Finally, regarding the relationship between CD133 expression and HIF-1α expression, in the luminal CD133 expression group in our study, the HIF-1α expression rate was lower, similar to that which was reported by Matumoto et al. [17]. However, in the cytoplasmic CD133 expression group, the HIF-1α expression rate was higher, similar to that reported for other organs. This finding suggests that for cytoplasmic CD133 expression with a high degree of malignancy, HIF-1α may upregulate its expression. In fact, in our study as well, the survival rate in gastric cancer in the HIF-1α expression group was lower. Even among cases in the HIF-1α expression group, in the group that was also CD133 positive, the survival rate was even lower. This result may have been because HIF-1α upregulated the cytoplasmic CD133 expression. However, in our study, even when the HIF-1α (+) CD133 (+) poor prognosis group was further classified based on CD133 expression type, no differences in survival rate based on expression type were observed. One possibility is that among the luminal CD133 expression group, those with HIF-1α expression also showed cytoplasmic CD133 expression.

Considering the relationship between CD133 and HIF-1α based on our study results, HIF-1α expression is increased with hypoxia, CD133 is expressed or retained in cancer cell cytoplasm, and tumor progression occurs as a result of this CSC-like function. In addition, release from this hypoxic state, namely via decreased HIF-1α, promotes the release of CD133 from the cytoplasm, which may then function in gland duct formation. If this is the case, then inhibiting HIF-1α expression may lead to improved prognosis in gastric cancer. However, before reaching this conclusion, it will be necessary to establish an accurate method for isolating the CD133 expression types and to conduct studies in gastric cancer cell lines using these isolated expression types.

## Conclusion

In our present study, gastric cancer with cytoplasmic CD133 expression was associated with lymph node metastases, peritoneal dissemination, chemotherapy resistance, recurrence, and poor prognosis. Evaluation of cytoplasmic CD133 expression in gastric cancer tissue sections may be useful in the future as a novel prognostic factor. Moreover, a significant correlation between HIF-1α expression and the CD133 immunohistochemical staining pattern was found.

## References

[1] Al-Hajj M, Clarke MF (2004). Self-renewal and solid tumor stem cells. Oncogene.

[2] Singh SK, Hawkins C, Clarke ID, Squire JA, Bayani J, Hide T (2004). Identification of human brain tumour initiating cells. Nature (Lond).

[3] Eramo A, Lotti F, Sette G, Pilozzi E, Biffoni M, Di Virgilio A (2008). Identification and expansion of the tumorigenic lung cancer stem cell population. Cell Death Differ.

[4] Ma S, Chan KW, Hu L, Lee TK, Wo JY, Ng IO (2007). Identification and characterization of tumorigenic liver cancer stem/progenitor cells. Gastroenterology.

[5] O’Brien CA, Pollett A, Gallinger S, Dick JE (2007). A human colon cancer cell capable of initiating tumour growth in immunodeficient mice. Nature (Lond).

[6] Ricci-Vitiani L, Lombardi DG, Pilozzi E, Biffoni M, Todaro M, Peschle C (2007). Identification and expansion of human colon cancer-initiating cells. Nature (Lond).

[7] Hermann PC, Huber SL, Herrler T, Aicher A, Ellwart JW, Guba M (2007). Distinct populations of cancer stem cells determine tumor growth and metastatic activity in human pancreatic cancer. Cell Stem Cell.

[8] Collins AT, Berry PA, Hyde C, Stower MJ, Maitland NJ (2005). Prospective identification of tumorigenic prostate cancer stem cells. Cancer Res.

[9] Chen YC, Hsu HS, Chen YW, Tsai TH, How CK, Wang CY (2008). Oct-4 expression maintained cancer stem-like properties in lung cancer-derived CD133-positive cells. PLoS One.

[10] Zhao P, Lu Y, Jiang X, Li X (2011). Clinicopathological significance and prognostic value of CD133 expression in triple-negative breast carcinoma. Cancer Sci.

[11] Hagiwara S, Kudo M, Ueshima K, Chung H, Yamaguchi M, Takita M (2011). The cancer stem cell marker CD133 is a predictor of the effectiveness of S1 + pegylated interferon α-2b therapy against advanced hepatocellular carcinoma. J Gastroenterol.

[12] Ong CW, Kim LG, Kong HH, Low LY, Lacopetta B, Soong R (2010). CD133 expression predicts for non-response to chemotherapy in colorectal cancer. Mod Pathol.

[13] Hayashi T, Ding Q, Kuwahata T, Maeda K, Miyazaki Y, Matsubara S (2012). Interferon-alpha modulates the chemosensitivity of CD133-expressing pancreatic cancer cells to gemcitabine. Cancer Sci.

[14] Soeda A, Park M, Lee D, Mintz A, Androutsellis-Theotokis A, McKay RD (2009). Hypoxia promotes expansion of the CD133-positive glioma stem cells through activation of HIF-1α. Oncogene.

[15] Sun C, Song H, Zhang H, Hou C, Zhai T, Huang L (2012). CD133 expression in renal cell carcinoma (RCC) is correlated with nuclear hypoxia-inducing factor 1α (HIF-1α). J Cancer Res Clin Oncol.

[16] Shimada M, Sugimoto K, Iwahashi S, Utsunomiya T, Morine Y, Imura S (2010). CD133 expression is a potential prognostic indicator in intrahepatic cholangiocarcinoma. J Gastroenterol.

[17] Matumoto K, Arao T, Tanaka K, Kaneda H, Kudo K, Fujita Y (2009). mTOR signal and hypoxia-inducible factor-1α regulate CD133 expression in cancer cells. Cancer Res.

[18] Japanese Gastric Cancer Association (2011). Japanese classification of gastric carcinoma. 3rd English edition. Gastric Cancer.

[19] Isobe T, Aoyagi K, Koufuji K, Shirouzu K, Kawahara A, Taira T, et al. Clinicopathological significance of hypoxia-inducible factor-1alpha (HIF-1α) expression in gastric cancer. Int J Clin Oncol. 2012 (Epub ahead of print). 10.1007/s10147-012-0378-8 22350022

[20] Horst D, Kriegl L, Engel J, Kirchner T, Jung A (2008). CD133 expression is an independent prognostic marker for low survival in colorectal cancer. Br J Cancer.

[21] Maeda S, Shinchi H, Kurahara H, Mataki Y, Maemura K, Sato M (2008). CD133 expression is correlated with lymph node metastasis and vascular endothelial growth factor-C expression in pancreatic cancer. Br J Cancer.

[22] Cheifec G, Capella C, Solcia E, Jao W, Gould V (1985). Amphicrine cells, dysplasias, and neoplasias. Cancer (Phila).

[23] Ishigami S, Ueno S, Arigami T, Uchikado Y, Setoyama T, Arima H (2010). Prognostic impact of CD133 expression in gastric carcinoma. Anticancer Res.

[24] Zhao P, Li Y, Lu Y (2010). Aberrant expression of CD133 protein correlates with Ki-67 expression and is a prognostic marker in gastric adenocarcinoma. BMC Cancer.

[25] Sasaki A, Kamiyama T, Yokoo H, Nakanishi K, Kubota K, Haga H (2010). Cytoplasmic expression of CD133 is an important risk factor for overall survival in hepatocellular carcinoma. Oncol Rep.

[26] Takahashi S, Kamiyama T, Tomaru U, Ishizu A, Shida T, Osaka M (2010). Frequency and pattern of expression of the stem cell marker CD133 have strong prognostic effect on the surgical outcome of colorectal cancer patients. Oncol Rep.

[27] Jao SW, Chen SF, Lin YS, Chang YC, Lee TY, Wu CC (2012). Cytoplasmic CD133 expression is a reliable prognostic indicator of tumor regression after neoadjuvant concurrent chemoradiotherapy in patients with rectal cancer. Ann Surg Oncol.

[28] Fukamachi H, Shimada S, Ito K, Ito Y, Yuasa Y (2011). CD133 is a marker of gland-forming cells in gastric tumors and ox17 is involved in its regulation. Cancer Sci.

[29] Yang J, Yan H, Hui L, Hui NL, Lei Z, Wei H (2012). Expressions of putative cancer stem cell markers ABCB1, ABCG2, and CD133 are correlated with the degree of differentiation of gastric cancer. Gastric Cancer.

